# Impaired mitochondrial β-oxidation in patients with chronic hepatitis C: relation with viral load and insulin resistance

**DOI:** 10.1186/1471-230X-13-112

**Published:** 2013-07-10

**Authors:** Chikako Sato, Takafumi Saito, Keiko Misawa, Tomohiro Katsumi, Kyoko Tomita, Rika Ishii, Hiroaki Haga, Kazuo Okumoto, Yuko Nishise, Hisayoshi Watanabe, Yoshiyuki Ueno, Sumio Kawata

**Affiliations:** 1 Department of Gastroenterology, Faculty of Medicine, Yamagata University, Yamagata 990-9585, Japan; 2 Nishinomiya Hyogo Prefectural Hospital, Hyogo 662-0918, Japan

**Keywords:** Ketogenesis, Fasting test, Hepatic steatosis, HCV

## Abstract

**Background:**

Hepatic steatosis is often seen in patients with chronic hepatitis C (CH-C). It is still unclear whether these patients have an impaired mitochondrial β-oxidation. In this study we assessed mitochondrial β-oxidation in CH-C patients by investigating ketogenesis during fasting.

**Methods:**

This study consisted of thirty patients with CH-C. Serum levels of insulin and hepatitis C virus (HCV) core protein were measured by chemiluminescence enzyme immunoassay. The subjects were then fasted, and venous blood samples were drawn 12 h and 15 h after the start of fasting. The levels of blood ketone bodies were measured by an enzymatic cycling method. The rate of change in total ketone body concentration was compared with that in eight healthy volunteers.

**Results:**

The rate of change in total ketone body concentration between 12 h and 15 h after the start of fasting was significantly lower in CH-C patients than in healthy volunteers (129.9% (8.5-577.3%) vs. 321.6% (139.6-405.4%); P <0.01). The rate of change in total ketone body concentration in patients with a serum level of HCV core protein of 10000 fmol/L or higher was significantly lower than in patients with a level of less than 10000 fmol/L (54.8% (8.5-304.3%) vs. 153.6% (17.1-577.3%); P <0.05). The rate of change in total ketone body concentration in patients with a homeostasis model assessment of insulin resistance (HOMA-IR) of 2.5 or higher was significantly lower than in patients with a HOMA-IR of less than 2.5 (56.7% (8.5-186.7%) vs. 156.4% (33.3-577.3%); P <0.01).

**Conclusions:**

These results suggest that mitochondrial β-oxidation is impaired, possibly due to HCV infection in patients with CH-C.

## Background

Hepatitis C virus (HCV) infection is a major cause of chronic liver injury. Hepatic steatosis is one of histologic features of chronic HCV infection with a risk of progression of liver diseases [1]. Hepatic steatosis is caused by some mechanisms, which include an increase of fatty acids uptake and synthesis, a decrease of fatty acids β-oxidation, or low level of secretion of very-low density lipoprotein. HCV core protein-transgenic mice develop hepatic steatosis due to impaired β-oxidation caused by mitochondrial damage [2]. However, there has been no evidence of impaired β-oxidation in patients with chronic hepatitis C (CH-C) *in vivo*.

During starvation, mitochondria produces acetyl CoA, which is converted into ketone bodies by fatty acids β-oxidation. In patients with impaired hepatic mitochondrial β-oxidation, ketogenesis is expected to be inadequate. Adult-onset type 2 citrullinemia (CTLN2) has been demonstrated to present as non-alcoholic fatty liver disease (NAFLD) [3]. CTLN 2 is associated with mutations in the SLC25A13 gene encoding citrin, which is a component of the mitochondrial malate-aspartate shuttle. Functional defectiveness of citrin impairs not only transport of aspartate from mitochondria but that of NADH into mitochondria. This induces activation of the citrate-malate shuttle with compensatory production of acetyl CoA, which in turn stimulates fatty acid synthesis. In addition, mitochondrial accumulation of malonyl CoA in a high NADH/NAD^+^ environment suppresses fatty acid oxidation. These circumstances lead to hepatic steatosis in patients with CTLN 2 [4]. Inui et al. have demonstrated that suppression of fatty acid oxidation is accompanied by impaired ketogenesis in such patients [5].

Based on this background, we measured the concentration of blood ketone bodies during fasting in order to evaluate mitochondrial β-oxidation in patients with CH-C, and thus to investigate a mechanism of steatosis associated with HCV infection. Here, we report for the first time that mitochondrial β-oxidation is impaired in patients with CH-C.

## Methods

### Patients

Thirty patients (14 male and 16 female, the mean age 54.2, ranging from 22 to 74 years old) with CH-C were studied. The patients were admitted to Yamagata University Hospital for treatment between March 2006 and May 2009. All of the patients had been positive for both serum anti-HCV and HCV RNA for more than 6 months, and had elevated levels of serum alanine aminotransferase (ALT). They were all negative for hepatocellular carcinoma, hepatitis B, autoimmune hepatitis, primary biliary cirrhosis, heart failure, renal insufficiency, a history of diabetes mellitus, excess alcohol intake (daily ethanol consumption >20 g) or drug abuse. None of the patients fulfilled the criteria for Metabolic Syndrome in Japan [6,7], i.e. the presence of at least two of the following three abnormalities in addition to visceral obesity (waist circumference: 85 cm or more in men, 90 cm or more in women): 1) triglycerides ≥150 mg/dl and/or HDL-cholesterol <40 mg/dl, or receiving treatment for this type of dyslipidemia; 2) systolic blood pressure ≥130 and/or diastolic blood pressure ≥85, or receiving treatment for hypertension; 3) fasting glucose ≥110 mg/dl or receiving treatment for diabetes. As a control group, eight volunteers (4 male and 4 female) were included (the mean age 30.5, ranging from 26 to 39 years old). All of them were healthy, with a BMI of <25 kg/m^2^, without medication or severe disease. Written informed consent to participate was obtained from all subjects, and the study protocol was approved by The Yamagata University Hospital Ethics Committee.

## Methods

### Clinical and laboratory data

Body height, weight and waist circumference were measured at the time of admission. Body mass index (BMI) was calculated as: BMI = body weight (kg)/body height (m)^2^ . Venous blood samples were collected after a 12-h overnight fast for standard biochemical testing and determination of serum insulin levels, HCV genotype and HCV core protein. Serum insulin was determined by chemiluminescence enzyme immunoassay (Lumipulseprestoinsulin^®^, Fujirebio Inc., Tokyo, Japan). Insulin resistance was determined based on the homeostasis model assessment of insulin resistance (HOMA-IR). HOMA-IR was calculated using the formula: [fasting glucose (mg/dl) × fasting insulin (μU/ml)] / 405 [8]. Insulin resistance was defined as HOMA-IR >2.5. The amount of HCV core antigen and HCV RNA in serum were measured by a chemiluminescence enzyme immunoassay (Lumispot Eiken HCV antigen^®^, Eiken Chemical Co., Ltd., Tokyo, Japan) and an amplicor HCV RNA detection kit (Amplicor HCV v2.0^®^, Roche Diagnostics, Tokyo, Japan) or a real-time PCR assay (COBAS^®^ TaqMan^®^ HCV Test, Roche Diagnostics, Tokyo, Japan), respectively.

### Fasting test

In general, ketone bodies are not detected during periods of feeding, but after the onset of fasting, glycogen is gradually consumed and ketone bodies are produced rapidly after about 12 hours of fasting. The rate of change in ketone body production between 12 and 15 hours represented the initial increase, and was interpreted as the initial velocity of ketogenesis.

Fasting tests were performed in both subjects and volunteers. They were permitted to drink water after their last meal, and blood samples were drawn to measure the proportion of ketone bodies, glucose, insulin, free fatty acid and triglyceride levels at 12 and 15 h after the last meal. Carnitine fractionation was also measured at 12 after the last meal, using an enzymatic cycling method (Total Carnitine Kainos^®^, Free Carnitine Kainos^®^, Kainos Laboratories, Inc., Tokyo, Japan). During fasting, urine organic acids were measured by gas chromatograph-mass spectrometry and then analyzed using software to determine the presence of disorders of organic acid metabolism. Serum ketone bodies were measured by an enzymatic cycling method (3-hydroxybutyrate Kainos^®^, Total ketone body Kainos^®^, ketone body standard reagent 2^®^, Kainos Laboratories, Inc. Tokyo, Japan). These measurements were performed by SRL Inc. (Tokyo, Japan). The rate of change in total ketone body concentration from 12 h to 15 h was calculated using the equation: (total ketone bodies at 15 h – total ketone bodies at 12 h)/ total ketone bodies at 12 h × 100.

### Histological assessment

Liver biopsies were performed under sonographic guidance in 25 patients who provided informed consent. The liver tissues were fixed in 10% formalin, embedded in paraffin and stained with hematoxylin-eosin and silver stain. Liver histology was graded and staged by a pathologist based on the international classification [9]. The grade of steatosis was modified as follows: grade 0 = no steatosis and between 0% and 5% of hepatocytes containing visible macrovesicular steatosis, grade 1 = between 5% and 33%, grade 2 = between 33% and 66%, and grade 3 = more than 66% according to the non-alcoholic fatty liver disease Activity Score [10].

### Statistical analysis

Results are expressed as the mean ± standard deviation (SD) or the median and range (in parenthesis). Student’s *t* test was used for normally distributed non-paired continuous variables. The rate of change in total ketone body concentration was assessed as a parametric value because the value was distributed parametrically after logarithmic transformation. Wilcoxon’s signed-ranks test was used for paired continuous variables. Comparisons between more than two groups were made by one-way analysis of variance. All P-values were based on a two-sided test of statistical significance. Differences at P <0.05 were considered to be statistically significant.

## Results

### Subject characteristics

The characteristics of thirty patients with chronic hepatitis C were shown in Table 1. The mean level of BMI was less than 25 kg/m^2^, and that of fasting plasma glucose was within normal range. The mean level of ALT was greater than the upper limit of normal range. Nine patients had a status of insulin resistance in whom HOMA-IR showed a level of 2.5 or greater. All patients were positive for HCV RNA in whom the mean level of serum HCV core antigen showed a 6505 fmol/L. Of the 30 patients, 20 (66.7%) were infected with the HCV genotype 1b, 6 (20.0%) with genotype 2a, and 4 (13.3%) with genotype 2b. Fifteen (60%) had a liver fibrosis grade of F1, 7 (28%) had F2, and 3 (12%) had F3. Six (24%) had a liver steatosis grade of 0, 18 (72%) had grade 1, 1 (4%) had grade 2, and none had grade 3.

**Table 1:** Patient characteristics

		**Patients**	**Reference value**
Age (yr) ^b^		54.2 ± 10.6	
Male/Female (ratio)		14/16 (0.47)	
BMI (kg/m^2^) ^b^		23.7 ± 2.7	18.5 - 25
ALT (IU/L) ^a^		47.5 (17–167)	8 - 42
γGTP (IU/L) ^a^		41 (13–152)	10 - 47
ChE (IU/L) ^b^		312.9 ± 88.2	185 - 431
Fasting plasma glucose (mg/dL) ^b^		95.5 ± 9.7	70 - 109
Insulin (μIU/mL) ^a^		8.7 (4.5 - 20.4)	1.84 - 12.2
HOMA-IR ^a^		2.0 (1.2 - 5.1)	
HOMA-IR > 2.5 (%)		9 (30)	
Triglyceride (mg/dL) ^b^		91.4 ± 31.1	30 - 149
Total cholesterol (mg/dL) ^b^		175.2 ± 25.4	129 - 219
Acylcarnitine (μmol/L) ^a^		9.25 (5.7 - 21.1)	6 - 23
HCV RNA (LogIU/mL) ^b^		6.1 ± 0.9	
HCV core antigen (fmol/L) ^a^		6505 (<20–23200)	
HCV Genotype	1b / 2a / 2b	20 / 6 / 4	
Inflammation	A0 / A1 / A2 / A3	0 / 15 / 10 / 0	
Fibrosis	F0 / F1 / F2 / F3 / F4	0 / 15 / 7 / 3 / 0	
Steatosis	G0 / G1 / G2 / G3	6 /18 / 1 / 0	

BMI, body mass index; ALT, alanine aminotransferase; γGTP, gamma-glutamyltranspeptidase; ChE, cholinesterase; HOMA-IR, homeostasis model assessment of insulin resistance.

Data are ^a^medians (min-max), ^b^the means ± standard deviation.

### Free fatty acids concentration during fasting in CH-C patients and healthy volunteers

**Figure 1:**
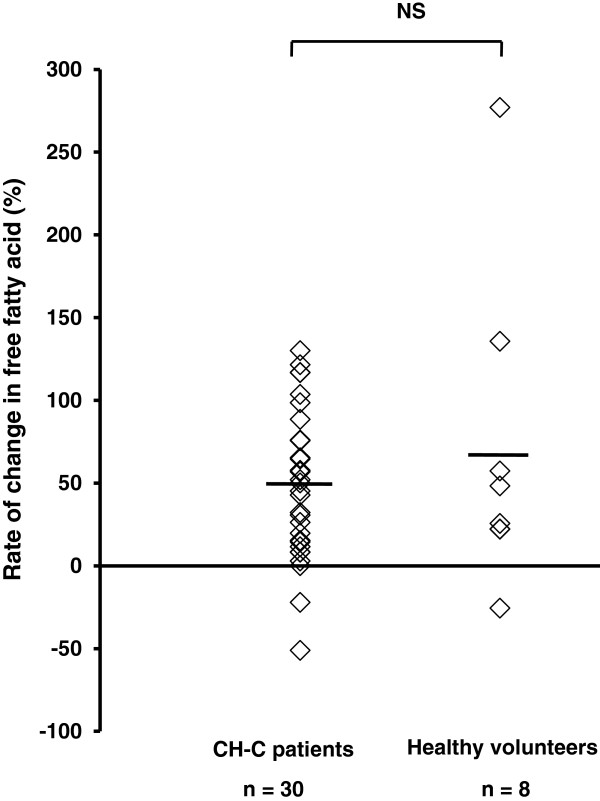
**Free fatty acids concentration during fasting in patients with chronic hepatitis C and healthy volunteers.** The rate of change in free fatty acid concentration between 12 h and 15 h after fasting was similar in both groups. The line represents a mean value. Welch’s *t*-test.

The concentration of free fatty acids increased by fasting in CH-C patients and healthy volunteers. The rate of change in free fatty acids concentration between 12 h and 15 h was similar in both groups (CH-C patients 48.6% ± 45.0 vs. healthy volunteers 70.3% ± 95.2; ns), as shown in Figure 1.

### Ketone body concentration during fasting in CH-C patients and healthy volunteers

**Figure 2:**
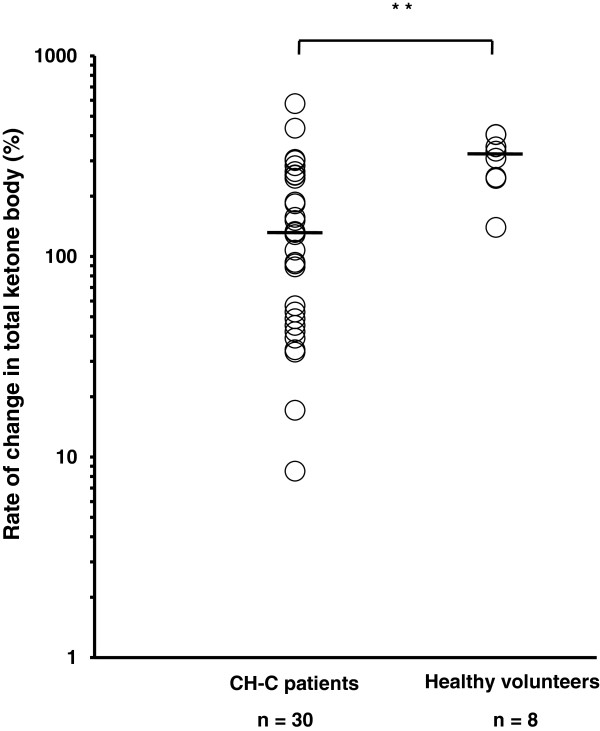
**Total ketone body concentration during fasting in patients with chronic hepatitis C and healthy volunteers.** The rate of change in total ketone body concentration between 12 h and 15 h after fasting was significantly lower in patients than in healthy volunteers (** denotes P <0.01). The line represents a median value. Log transformation was performed. Student’s *t* test.

**Figure 3:**
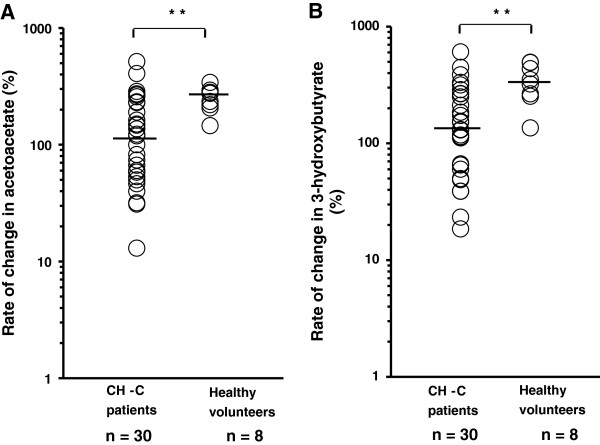
**Ketone body fraction concentration during fasting in patients with chronic hepatitis C and healthy volunteers.** The rates of change in both acetoacetate **(A)** and 3-hydroxybutyrate **(B)** between 12 h and 15 h after fasting were significantly lower in patients than in healthy volunteers (** denotes P <0.01). The line represents a median value. Log transformation was performed. Student’s *t* test.

The levels of total blood ketone bodies were elevated in both CH-C patients and healthy volunteers 15 h after the start of fasting, but the rate of change between 12 h and 15 h after fasting was significantly different between them. The rate of change in total ketone body concentration between 12 h and 15 h was shown in Figure 2. It was significantly lower in CH-C patients than in healthy volunteers (129.9% (8.5-577.3%) vs. 321.6% (139.6-405.4%); P <0.01). The rates of change in both acetoacetate (Figure 3A) and 3-hydroxybutyrate (Figure 3B) between 12 h and 15 h after fasting were also significantly lower in CH-C patients than in healthy volunteers (acetoacetate : 109.5% (-5.8-514.3%) vs. 254.8% (145.5-341.7%) , 3-hydroxybutyrate : 130.8% (-3.8-606.7%) vs. 337% (135.5-495%) ; P <0.01).

**Figure 4:**
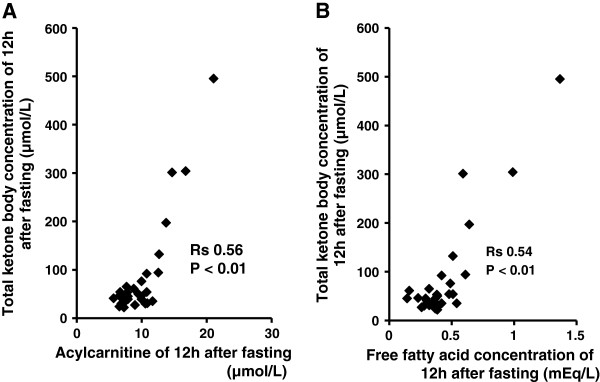
**Ketone body concentration are related with acylcarnitine and free fatty acid in patients with chronic hepatitis C.** There is a significant positive correlation between the concentration of total ketone body and the levels of acylcarnitine (rs 0.56, P <0.01),**(A)**, as well as free fatty acids (rs 0.54, P <0.01), **(B)**.

There was a significant positive correlation between the concentration of total ketone body and the levels of acylcarnitine (rs 0.56, P <0.01) at 12 h after fasting, as shown in Figure 4A, similar to the pattern of free fatty acids (rs 0.54, P <0.01, Figure 4B). The level of acylcarnitine was significantly lower in CH-C patients than in healthy volunteers (9.25 μmol/L (5.7-21.1 μmol/L) vs. 11.65 μmol/L (9.3-17 μmol/L); P <0.05).

### Relationship between the rate of change in total ketone body concentration and clinical parameters in CH-C patients

**Figure 5:**
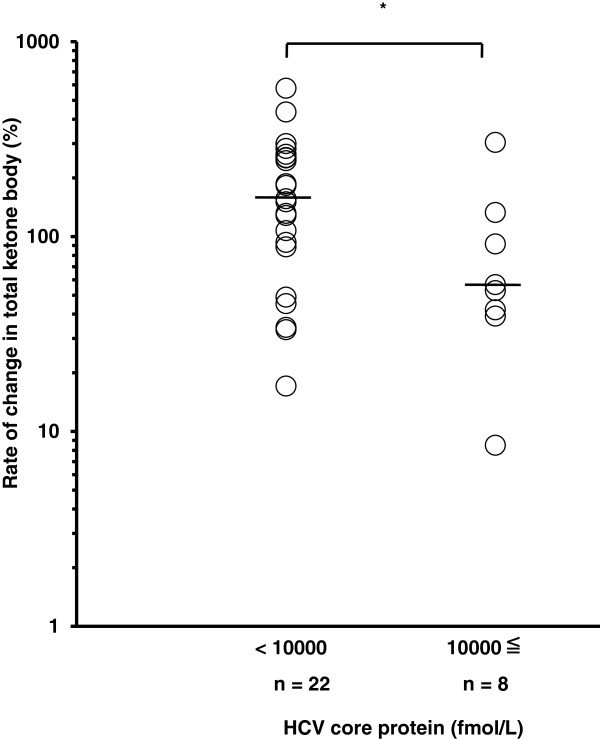
**HCV core protein and the change in total ketone body concentration during fasting.** The rate of change in total ketone body concentration between 12 h and 15 h after fasting in patients with a serum HCV core protein level of 10000 fmol/L or higher was significantly lower than that in patients with a level of less than 10000 fmol/L (* denotes P <0.05). The line represents a median value. Log transformation was performed. Student’s *t* test.

**Figure 6:**
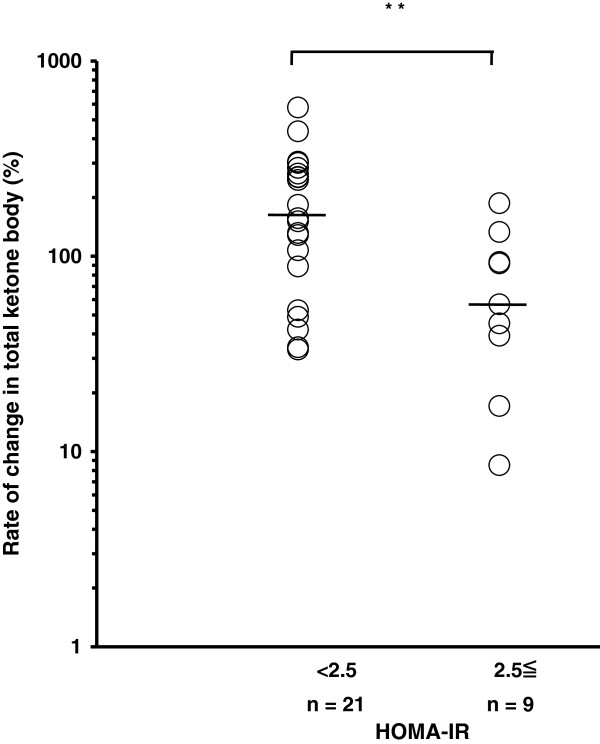
**Insulin resistance and the change in total ketone body concentration during fasting.** The rate of change in total ketone body concentration in patients with a higher HOMA-IR value (2.5 or greater) was significantly lower than that in patients with a value of less than 2.5 (** denotes P <0.01). The line represents a median value. Log transformation was performed. Student’s *t* test.

**Figure 7:**
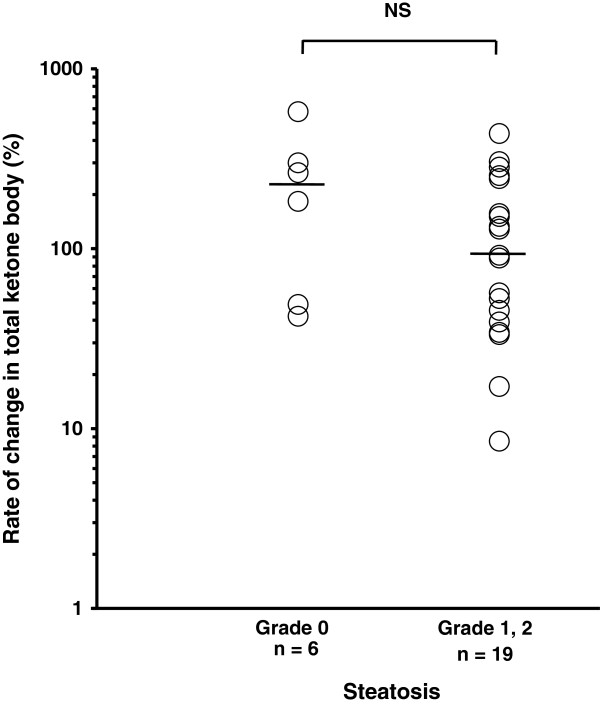
**Hepatic steatosis and the change in total ketone body concentration during fasting.** The patients with steatosis had a relatively low rate of change in total ketone body concentration between 12 h and 15 h after fasting in comparison with those without steatosis, although it was not significant. The line represents a median value. Log transformation was performed. Student’s *t* test.

We stratified CH-C patients into two groups based on the clinical parameters. The rate of change in total ketone body concentration between 12 h and 15 h in patients with a serum HCV core protein level of 10000 fmol/L or higher was significantly lower than that in patients with a level of less than 10000 fmol/L (54.8% (8.5-304.3%) vs. 153.6% (17.1-577.3%); P <0.05) (Figure 5). In addition, the rate of change in total ketone body concentration in patients with a higher HOMA-IR value (2.5 or greater) was significantly lower than that in patients with a value of less than 2.5 (56.7% (8.5-186.7%) vs. 156.4% (33.3-577.3%); P <0.01) (Figure 6). The patients with biopsy-proven steatosis had a relatively low rate of change in total ketone body concentration between 12 h and 15 h in comparison with those without steatosis, although the rate was not significantly different between them (Figure 7). There was no significant difference in the rate of change in total ketone body concentration among the HCV genotypes (1b 120.2% (8.5-577.3%), 2a 129.9% (91.7-304.3%), 2b 135.8% (56.7-253.3%)). No significant difference in the rate of change in total ketone body concentration was demonstrated among the stages of fibrosis (F1 91.7% (17.1-436%), F2 133% (8.5-283.3%), F3 88.6% (34.2-577.3%)).

## Discussion

Hepatitis C virus (HCV) is the leading cause of chronic hepatitis, subsequent liver cirrhosis and hepatocellular carcinoma. Hepatic steatosis is commonly seen in patients with chronic HCV infection having a high viral load, and it is in part associated with the development of insulin resistance [1], hepatic fibrosis [11] and hepatocarcinogenesis [12] during infection. Steatosis is also associated with a lower rate of sustained response to anti-viral therapy [13], and shows improvement after successful eradication of HCV by anti-viral therapy [14].

In general, fat accumulation in hepatocytes can result from several causes; increase of fatty acid uptake by hepatocyte, increase of fatty acid synthesis in hepatocyte, decrease of hepatic fatty acid oxidation, decrease of very-low density lipoprotein secretion. The mechanisms of steatosis in HCV infection are not fully understood. In the previous study using liver biopsy specimens of patients with HCV infection, it is shown that expression of peroxisome proliferator-activated receptor (PPAR)-α is impaired, which is an important factor in the regulation of mitochondrial β-oxidation [15]. Therefore, impaired mitochondrial β-oxidation is supposed to be a mechanism of hepatic steatosis observed in the state of HCV infection.

However, there is no previous study which investigated whether mitochondrial β-oxidation is impaired in patients with CH-C *in vivo*. In the present study, therefore, we focused on the mechanism of ketogenesis in humans by investigating ketogenic capacity during fasting. The rate of change in total ketone body concentration between 12 h and 15 h after the start of fasting was significantly lower in CH-C patients than in healthy volunteers, while the rate of change in free fatty acids concentration was similar in both groups. Therefore there is a possibility that steps from acetyl-CoA to ketone bodies are impaired in patients with CH-C. In addition, Hoppel et al. reported that acylcarnitine increased during fasting and ketone bodies correlated with short-chain acylcarnitines. It is speculated that the increase in short-chain acylcarnitines may be a by-product of fatty acid β-oxidation [16]. In our patients, the level of acylcarnitine was significantly lower in CH-C patients than in healthy volunteers. Thus, these support that mitochondrial β-oxidation is impaired in patients with CH-C. Further studies are needed to assess which step is involved in the impairment of ketone bodies formation in HCV infection.

During starvation, ketone bodies increase in the body under conditions of normal mitochondrial β-oxidation. Since insulin secretion decreases during fasting, synthesis of triglyceride from acyl CoA is suppressed. Therefore, acyl CoA is β-oxidized to acetyl CoA in mitochondria. Oxaloacetate is used for gluconeogenesis during fasting. Under this condition, acetyl CoA cannot conjugate oxaloacetate, and the tricarboxylic acid (TCA) cycle is inhibited. Inhibition of the TCA cycle also occurs through consumption of nicotinamide adenine dinucleotide (NAD^+^) and the production of reduced nicotinamide adenine dinucleotide (NADH) via β-oxidation. Consequently, acetyl CoA shifts towards ketogenesis. Acetyl CoA enters the TCA cycle and is used as fuel in muscle. Thus, the liver is the only organ that produces ketone bodies and secretes them into blood. In individuals with impaired hepatic mitochondrial β-oxidation, it is expected that ketogenesis would not be adequate. This is a reason why measurement of blood ketone body concentration in a fasting state facilitates assessment of mitochondrial β-oxidation *in vivo*[5].

In the present study, the rate of change in total ketone body concentration in patients with a serum level of HCV core protein of 10,000 fmol/L or higher was significantly lower than in patients with a level of less than 10,000 fmol/L, showing that patients with a higher level of serum HCV core protein had lower ketogenic capacity. HCV core protein induces hepatic steatosis with disappearance of the double structure of mitochondrial membranes in HCV core transgenic mice [2]. HCV core protein is largely associated with mitochondrial dysfunction [17]. Moreover, recent studies have reported that HCV core protein downregulates the expression of PPAR-α, which is abundant in hepatocytes and is an important factor in the regulation of mitochondrial β-oxidation [15,18]. Our data suggest an impairment of mitochondrial β-oxidation by HCV infection.

Although no significant relationship between fatty acid oxidation and the grade of steatosis was demonstrated in this study (Figure 7), this issue would be worth investigating in a larger cohort of patients. HCV infection induces mitochondrial dysfunction as a result of oxidative stress, which is closely related to liver inflammation and hepatocarcinogenesis [19]. Oxidative stress is associated with impairment of fatty acid oxidation, and thus impaired ketogenesis seems to represent the increased oxidative stress in CH-C patients.

Insulin resistance in patients with CH-C has been reported [20]. At this study, insulin resistance, HOMA-IR >2.5, was observed in 9 of 30 patients. In this study, a significant positive correlation was evident between the concentration of total ketone bodies and that of free fatty acids. However, in some patients with insulin resistance, the concentrations of both free fatty acids and ketone bodies were not so high. The rate of change in the concentrations of total ketone bodies was significantly lower in patients with a higher HOMA-IR value (2.5 or greater) than in those with a value of less than 2.5. Many other factors may influence the level of fatty acid. Further studies are needed to elucidate the mechanism of insulin resistance in CH-C patients.

Our CH-C patients were significantly older than the healthy volunteers. However, we did not observe any significant correlation between the age of our subjects and the rate of change in total ketone body concentration within the age range investigated (data not shown). Elderly people in good health have a similar capacity to produce ketones to middle-aged or young adults [21].

## Conclusions

The results of our study suggest that mitochondrial β-oxidation is impaired, possibly due to HCV infection. Further studies are needed to elucidate the detailed pathophysiology of impaired fatty acid metabolism in CH-C and its clinical significance.

## Abbreviations

CH-C: Chronic hepatitis C; HCV: Hepatitis C virus; HOMA-IR: Homeostasis model assessment of insulin resistance; CTLN2: Adult-onset type 2 citrullinemia; NAFLD: Non-alcoholic fatty liver disease; ALT: Alanine aminotransferase; BMI: Body mass index; SD: Standard deviation; PPAR: Peroxisome proliferator-activated receptor; TCA: Tricarboxylic acid; NAD: Nicotinamide adenine dinucleotide; NADH: Reduced nicotinamide adenine dinucleotide; SREBP: Sterol regulatory element-binding protein.

## Competing interests

No financial interests to disclosure that related to this study.

## Authors’ contributions

SC, MK, KT, TK, IR, HH, OK and NY contributed to data collection and data analysis. SC, ST, WH and UY contributed to data interpretation and manuscript writing. KS contributed to the design and conduct of the study. All authors read and approved the final manuscript.

## Pre-publication history

The pre-publication history for this paper can be accessed here:

http://www.biomedcentral.com/1471-230X/13/112/prepub
